# The Influence of Standard and Freeze-Dried Tofu on the Phenols and Quality of Virgin Olive Oil Used as an Immersion Medium

**DOI:** 10.3390/molecules30030672

**Published:** 2025-02-03

**Authors:** Olivera Koprivnjak, Valerija Majetić Germek, Paula Žurga, Karolina Brkić Bubola

**Affiliations:** 1 Department of Food Technology and Quality Control, Faculty of Medicine, University of Rijeka, Braće Branchetta 20, 51000 Rijeka, Croatia; olivera.koprivnjak@uniri.hr; 2 Teaching Institute of Public Health of Primorsko-Goranska County, Krešimirova 52a, 51000 Rijeka, Croatia; paula.zurga@zzjzpgz.hr; 3 Institute of Agriculture and Tourism, Karla Huguesa 8, 52440 Poreč, Croatia

**Keywords:** virgin olive oil, tofu, phenols, sensory properties

## Abstract

Various protein-rich foods are traditionally immersed in virgin olive oil (VOO), a medium rich in phenols, which are health-promoting and sensorially important compounds. Immersing tofu in VOO may modify the sensory properties and nutritional value of both due to the oil’s hydrophilic phenol interactions with proteins and water. In this study, cubes of fresh tofu (T) (70% water) and freeze-dried tofu (FD-T) (5% water) were immersed in VOO for 7 days of cold storage. The changes in the phenolic compound content and standard quality parameters of the oil were noted after 1, 3, 5, and 7 days of contact with the tofu. The total phenols in the oil were determined using the Fast Blue BB assay, while single phenols were analyzed by HPLC-UV/VIS. During the 7 days, the total phenols in the oil decreased by up to 56% and 26% under the influence of fresh and freeze-dried tofu, respectively, including a significant decrease in hydroxytyrosol, oleacein, tyrosol, and oleocanthal. The water content and its release from fresh tofu significantly contributed to this decline. The degradation of the quality of the oil in contact with the fresh tofu was observed only in its sensory properties, with a marked reduction in the intensity of its fruitiness, bitterness and pungency.

## 1. Introduction

Tofu is a traditional unfermented product derived from soybeans (*Glycine max*) that is consumed as part of vegetarian, vegan, and low-calorie diets. It is an excellent plant-based protein source [1] and contains bioactive compounds such as isoflavones, saponins, phytosterols, and peptides [2]. Tofu is produced from previously soaked soybeans ground with water. The filtered suspension is boiled, and coagulants (commonly, salts such as calcium sulfate or acids like glucono-δ-lactone) are added to induce curd formation. The curd is then collected, pressed, and shaped into white blocks of varying firmness. The texture, flavor, and nutritional value of tofu are influenced by the thermal denaturation and coagulation properties of soy proteins [3]. According to Yasin et al. [4], the average nutritional composition of tofu produced from eight soybean varieties with acidic coagulation is 55% crude protein, 31% crude fat, 2% total ash (based on dry mass), and 73% moisture. Soybean proteins are high-quality proteins with relatively good digestibility, which is comparable to animal proteins, and have a well-balanced composition of amino acids, though they are limited in sulfur-containing ones such as methionine and cysteine [1].

Because of its soft, cheese-like structure, which is compact and firm enough for cutting, pieces of tofu can be immersed in virgin olive oil (VOO) primarily to improve their nutritional and sensory properties, considering that this type of preservation is not a common practice. Similarly, other foods, such as various cheeses, fish, and vegetables are preserved in VOO, during which interactions and the transfer of substances between the food and oil take place [5,6,7]. In the aforementioned studies, contact between the food and oil resulted in a noticeable decrease in the phenol content of VOO. Additionally, Castillo-Luna and Priego-Capote [5] established a significant enrichment of cheese, salmon, cod, tomatoes, and eggplants with VOO phenols (mainly tyrosol, hydroxytyrosol, and oleuropein aglycone) after 30 days of immersion in VOO. Studies by Majetić Germek et al. [7] on whey cheese and tofu, as well as those by Castillo-Luna and Priego-Capote [5] on soft and hard cheese, indicate that the transfer of phenols from VOO into these foods probably occurs to the greatest extent within a short contact period. However, the dynamics of phenol transition during the first days of contact between VOO and protein-rich foods remain poorly researched. Therefore, in the present research, the impact of high-moisture and protein-rich food on the phenolic compounds of VOO during the first seven days of contact is examined in more detail.

Phenols can interact and form complexes with proteins, influencing the structure and functionality as well as nutritional and sensory properties of both [8,9,10]. Secoiridoid phenols contribute to the characteristic bitterness and pungency of VOO. Among secoiridoids, oleuropein aglycones are both bitter and pungent, some of which express strong bitterness, while ligstroside aglycones such as oleocanthal greatly contribute to pungency [11]. A study by Peyrot des Gachons et al. [9] confirmed that egg yolk and whey proteins suppress the bitterness and pungency of VOO in model systems. Given the significant contribution of phenolic substances to the desirable flavor properties of VOO, the research also included monitoring the sensory properties of the oil, which is a novelty compared to previous similar studies.

Protein–phenol interactions can be non-covalent or covalent, depending on the physicochemical conditions (pH, temperature, moisture, ionic strength, phenol/protein ratio, and the presence of oxygen, oxidants, and antioxidants) and the structural features of proteins and phenols [12]. Non-covalent interactions include hydrophilic and hydrophobic interactions, whereas covalent bonds primarily occur through phenol oxidation and the formation of quinones, which can react with nucleophilic groups in protein side chains. Covalent phenol–protein bonding may stabilize phenols against degradation in the gastrointestinal tract but also may influence their metabolic fate and bioavailability [13,14]. The moisture content in food systems may influence protein–phenol interactions and bonding types, though the extent and details of the mechanisms of this remain unclear [12]. The potential of high moisture to induce phenolic oxidation (autooxidation or enzymatic oxidation) could contribute to covalent bonding [15].

This study reveals, for the first time, the dynamics of single and total phenol changes in VOO during the initial contact period with protein-rich food, represented by tofu. Considering the hydrophilic nature of phenolic compounds [16], the role of water in food was investigated by monitoring the phenol changes in VOO as an immersion medium for freeze-dried and fresh tofu under identical conditions. Furthermore, the enrichment of defatted FD-T with VOO phenolic compounds and the sensory attributes of VOO after short contact with fresh tofu were examined for the first time.

## 2. Results and Discussion

Considering the current, although still limited, knowledge that phenols from VOO likely migrate to the greatest extent into protein-rich foods in a relatively short contact time [5,7], four time points (1, 3, 5, and 7 days) were selected for the 7-day experiment. In the parallel monitoring of fresh tofu (70% of water) and FD-T (5% of water), identical contact conditions were maintained. This was achieved by ensuring that the pieces of tofu obtained by freeze-drying had the same dimensions as those of fresh tofu and that the removal of water from the FD-T to the level of bound water did not result in protein denaturation or the degradation of other ingredients. Additionally, an equal ratio of the dry matter mass of tofu to VOO was ensured by filling the jars with the same number of tofu pieces (60 per jar) and the same mass of oil (100 g of oil per jar). A storage temperature of 6 ± 2 °C was chosen as a compromise between conditions that may delay microbiological spoilage of tofu and those that prevent the solidification of the oil.

### 2.1. Total Phenols and Single Phenolic Compounds in VOO

**Figure 1:**
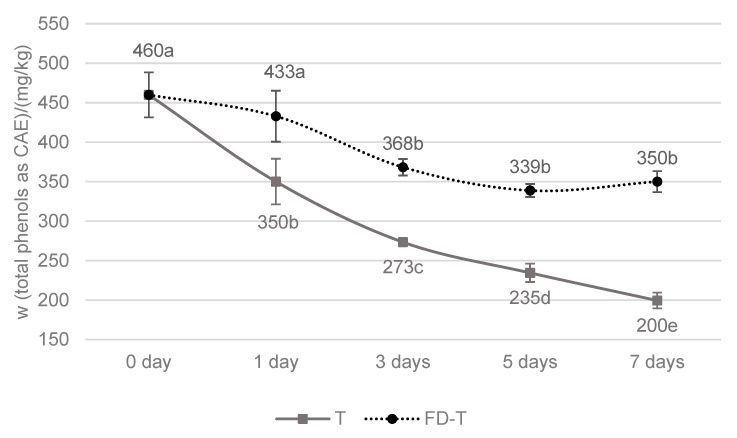
The mass fraction of total phenols in VOO used for dipping fresh tofu (T) and freeze-dried tofu (FD-T) under cold storage (6 ± 2 °C) determined by the Fast Blue BB test. The results are presented as the mean value ± standard deviation of 3 sample preparations. Mean values labeled with different letters are statistically different (one-way ANOVA, Tukey’s test for unequal N, *p* < 0.05) among immersion time point within the same type of VOO sample. CAE—caffeic acid equivalents; 0 day—control extra VOO that was not in contact with tofu.

From the data presented in Figure 1, it is evident that the control extra VOO sample contained a relatively high proportion of total phenols and that the value measured on the seventh day of contact with the fresh tofu decreased by approximately 56% compared to the control sample.

The total phenol values measured at each time point of the experiment decreased continuously and significantly. The most pronounced decrease (approximately by 24% compared to the control sample) was observed already after the first day of contact with fresh tofu. A similar outcome was reported in the research by Majetić Germek et al. [7], where one day of contact between fresh tofu and VOO resulted in an even more pronounced decrease in the total phenols (by 37%). In the studies by Klisović et al. [6] and Castillo-Luna and Priego-Capote [5], the total phenol content in VOO was not monitored during the early phase of contact with cheese or whey cheese but was instead measured after 30 days. By this time, the decrease in the phenols in VOO was already extensive, ranging from 83% for whey cheese to 98% for hard cheese. Castillo-Luna and Priego-Capote [5] attributed such a decrease to the combined effects of two mechanisms: (1) the migration of hydrophilic phenols from oil to food, driven by the water in food, and (2) the loss of phenols due to the antioxidant action of these compounds.

The major loss of phenols in oxidation reactions under the conditions in which this research was conducted (short duration, low storage temperature, darkness, and minimal oxygen exposure) is, however, very unlikely. Klisović et al. [6] suggest that changes in the composition of VOO should primarily be attributed to the migration of substances between the two matrices. The migration of water from fresh tofu into the oil was observed as water inclusions that gradually formed a water layer at the bottom of the jar. Considering the high affinity of VOO phenols for the aqueous phase, it is highly probable that a significant proportion of VOO phenols migrated into the water inclusions thus formed. This is further supported by the significantly lower impact of FD-T on the decrease in the total phenols in the VOO compared to the impact of fresh tofu at all the time points of the experiment (Figure 1).

On the first day of contact with the FD-T, the reduction in the total phenols in the VOO was not significant, amounting to only 6% compared to the control sample. Considering the high protein content in the dry matter of tofu (61%, according to the manufacturer’s nutritional label), the probability of close encounters between phenolic and protein molecules (and, thus, their interactions) should be high in FD-T. Quintero-Flórez et al. [17] reported a very rapid reaction (within one minute) between phenols from VOO and protein mucin in an aqueous solution. In light of this, the findings of our research suggest that interactions between phenols and proteins are significantly slowed in media such as oil and freeze-dried materials that contain minimal water. The water content in the FD-T was extremely low (approximately 5%), consisting primarily of bound water. In the FD-T variant, there was practically no migration of water from the material into the oil. A significant reduction in the total phenol mass fraction in VOO as the immersion medium was recorded only on the third day of FD-T–oil contact. At the next two time points of the experiment, the total mass fraction of phenols remained stable (in the range of 20–26%).

Consistent with the changes in the total phenols determined using the Fast Blue BB method, the mass fraction of most single phenolic compounds (except vanillic acid and vanillin) significantly decreased after the first day of contact with fresh tofu and, to a considerably lesser extent, after contact with FD-T (Table 1 and Table 2). In both tofu variants, oleocanthal and oleacein contributed the most to this reduction, as they were the two most abundant phenols identified in the VOO used in this research. However, the greatest reduction in the mass fraction due to contact with fresh tofu was observed for phenolic alcohols, specifically tyrosol and hydroxytyrosol (Table 2). These compounds are more soluble in water compared to the other analyzed phenolic compounds. Nevertheless, the data in Table 2 reveal that the solubility of different phenolic compounds in water does not correlate with the percentage of their mass fraction reduction in VOO under the influence of fresh tofu. This observation supports the hypothesis that complex interactions occur between VOO phenols and ingredients of food immersed in oil. For example, a high moisture content has the potential to promote covalent protein–phenol interactions [12] by inducing the autoxidation or enzymatic oxidation of phenols to quinones, which are more reactive toward proteins [15].

In the FD-T variant, the effect of water on phenol reduction in VOO was eliminated. As expected, the reduction in the mass fraction of single phenols was less pronounced in this variant. The contribution of water or other tofu components to the decline in the phenolic content of VOO is represented in Table 2 by the ratio of the percentage reduction caused by the fresh tofu to that caused by the FD-T. It can be reasonably considered that a higher ratio indicates a greater contribution of water. This ratio was highest for tyrosol and pinoresinol and lowest for oleacein. Furthermore, a distinct trend in the h factor (the ratio between hydroxytyrosol and tyrosol conjugated compounds) was observed between the two tofu variants during the seven-day contact period. In the fresh tofu variant, this ratio remained constant, whereas in the FD-T variant, it continuously decreased. This finding suggests that hydroxytyrosol conjugated compounds were more susceptible to reactions with tofu ingredients other than water.

It should be noted that the conditions of contact between oil and T and oil and FD-T were not entirely identical. Specifically, FD-T is a porous material, which is not the case with fresh tofu. Consequently, it can be reasonably assumed that the contact surface of the FD-T with oil was greater compared to that of the fresh tofu. Therefore, a decrease in the total phenols in the range of 20–26% in the VOO in contact with the FD-T can be attributed to two factors with opposing influences. The large contact surface facilitates more interactions between phenols and proteins (contributing to a reduction in the total phenols in the VOO), whereas the low water content and absence of water inclusions promote the retention of phenols in the VOO. In other words, the differences in the effect of T and FD-T would likely be even greater than observed if it were possible to achieve an identical contact surface between these two tofu variants and the oil.

### 2.2. Total Phenols in Freeze-Dried Tofu

**Figure 2:**
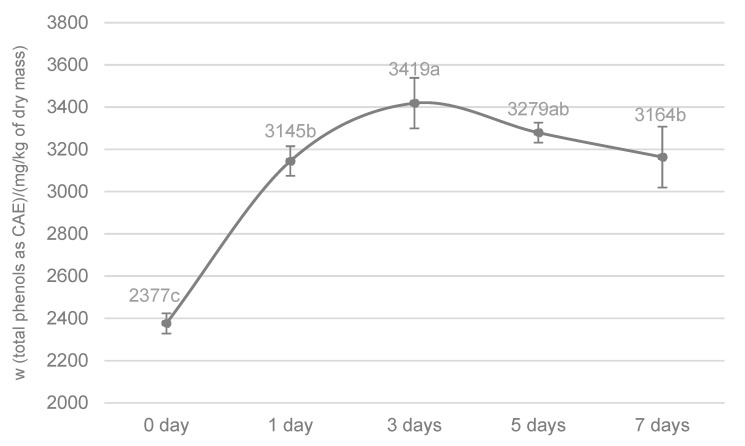
The mass fraction of total phenols in defatted freeze-dried tofu (FD-T) immersed in VOO under cold storage (6 ± 2 °C) determined by the Fast Blue BB test. The results are presented as mean value ± standard deviation of 3 sample preparations. Mean values labeled with different letters are statistically different (one-way ANOVA, Tukey’s test for unequal N, *p* < 0.05) among immersion time points. CAE—caffeic acid equivalents; 0 day—control FD-T that was not in contact with VOO.

The mass ratio of total phenols in tofu immersed in oil was monitored only for the defatted freeze-dried variant. Fresh tofu was not considered due to challenges associated with adequately removing the absorbed oil and water from the pieces separated from the infusion prior to the extraction of phenolic compounds. The data presented in Figure 2 indicate that the native FD-T contained a significant proportion of phenols extractable with a 70% ethanol solution.

Yin et al. [26] reported that tofu contains both soluble and insoluble fractions of phenolic substances. The soluble fraction is predominantly composed of isoflavones in the form of glucosides, with a smaller portion comprising phenolic acids such as vanillic acid and syringic acid. In the aforementioned study, Yin et al. [26] determined the content of soluble phenols in tofu to be approximately 900 mg gallic acid equivalents per kilogram of dry matter, whereas in the present study, this value was about 2400 mg caffeic acid equivalents. This discrepancy may arise from real differences in the composition of the samples or from variations in the methods used for the extraction and determination of the soluble phenols. Yin et al. [26] applied methanol and ethyl acetate for extraction and used the Folin–Ciocalteu reagent for phenol quantification. A significant increase in the total hydrophilic phenol content of FD-T was observed after the first day of contact with the VOO (a 32% increase compared to the control sample) and on the third day (a 44% increase). Thereafter, the values remained largely stable, suggesting that most of the available covalent binding sites for phenols in FD-T were likely saturated. Also, such a dynamic equilibrium may be established between the freeze-dried material and the oil due to the continuous formation and disruption of non-covalent hydrophilic and hydrophobic protein–phenol interactions [12]. As a confirmation of these observations, the trend and dynamics of the phenol decrease in the VOO in contact with the FD-T were closely aligned with the trend and dynamics of the phenol increase in the FD-T (Figure 1). The data from the present study are not directly comparable with those of Castillo-Luna and Priego-Capote [5], as the cheese samples in their study were not defatted prior to the extraction of hydrophilic phenols, and the results were expressed per unit mass of material without accounting for the fat and water content.

### 2.3. Quality Indices and Sensory Attributes of VOO

Physicochemical quality indicators of VOO (Table 3) were determined on the first and last day of the experiment, as no significant changes in these indicators were expected during shorter intervals of contact between the oil and tofu. The peroxide value remained largely unchanged; however, a significant decrease was observed in another indicator of primary oxidation products (K_232_), as well as secondary oxidation products (K_268_). Majetić Germek et al. [7] reported almost identical results for the seven-day storage of fresh tofu in contact with VOO. Similar findings were documented by Klisović et al. [6], who observed a significant decrease in K_232_ and K_268_ after 30 days of storing whey cheese in VOO, accompanied by a significant increase in the peroxide value.

The proportion of free fatty acids also decreased significantly, although the reduction was negligible in relation to the upper limit for the extra VOO category [27]. For this quality indicator, the findings also aligned with those of Majetić Germek et al. [7], who suggested that both free fatty acids and fatty acid oxidation products, being polar substances, are capable of migrating into water inclusions within oil, similar to hydrophilic phenols. The results obtained in this study for the FD-T variant, where no water inclusions in the oil were formed, support their assumption: over the seven-day period, no significant differences were detected in the proportion of free fatty acids and K_232_, while the K_268_ value increased significantly.

Due to contact with fresh tofu, sensory defects may arise in VOO. In addition, research by Peyrot des Gachons et al. [9] demonstrated that the presence of protein in food can change the taste perception due to interactions between the protein and oleocanthal (responsible for the pungent throat sensation) as well as the bitter compounds of VOO. Pripp et al. [10] described the interactions of phenols extracted from VOO and milk proteins as weak in the case of secoiridoid phenols (including compounds such as oleocanthal and oleuropein) and very weak or non-existent in the case of simple phenols (e.g., tyrosol and hydroxytyrosol). However, given that the structure of soy proteins differs from that of milk proteins, and considering the significant decrease in hydrophilic phenols in VOO in contact with fresh tofu (Figure 1), it was hypothesized that the intensity of VOO’s flavor properties would be reduced under the conditions of this study.

**Figure 3:**
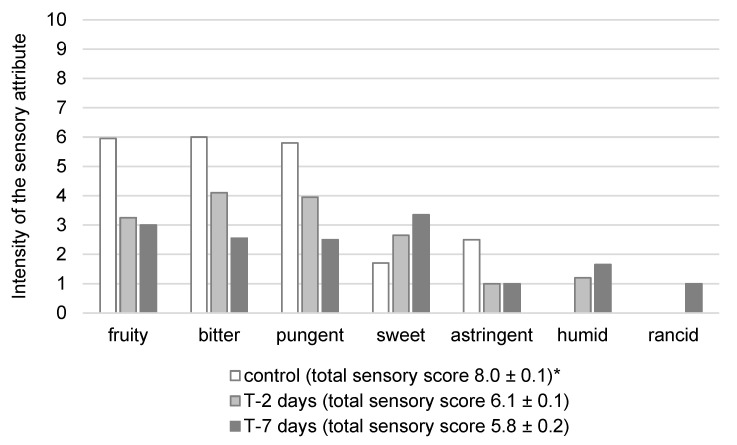
Intensity of the sensory attributes of VOO used for immersion of fresh tofu (T) under cold storage (6 ± 2 °C). The results are presented as median of 8 values given by assessors. * Total sensory score (1 point—the lowest quality; 9 points—the highest quality) represents mean value ± standard deviation of 8 values given by assessors.

As shown in Figure 3, after only two days of contact between VOO and fresh tofu, there was a significant decrease in the intensities of bitterness, pungency, and astringency, accompanied by an increase in sweetness. After seven days of contact, the intensity reductions in bitterness, pungency, and astringency were even more pronounced, resulting in the oil transitioning from its initial medium intensity (ranging from 3.1 to 6.0 on the linear scale) to a delicate intensity (≤ 3.0) [27]. Genovese et al. [28] classified VOOs according to the total phenol content into three categories of bitterness and pungency intensity, defining oils with 220–340 mg/kg of total phenols as medium intensity and oils containing less than 220 mg/kg as almost imperceptible intensity. The results of the sensory analysis of VOO (Figure 3) and the total phenol content in VOO (Figure 1) align with the criteria proposed by Genovese et al. [28]: after two days of contact, the oil contained approximately 300 mg/kg of total phenols (medium intensity of bitterness and pungency), while after seven days, the total phenol content decreased to approximately 200 mg/kg (delicate intensity or almost imperceptible bitterness and pungency).

In addition to the intensity decrease of the taste properties, the sensory analysis also revealed a reduction in the intensity of desirable flavor attributes (fruitiness), which declined from an initial medium intensity to a delicate intensity after seven-day contact. Furthermore, slightly perceptible undesirable sensory properties were detected after 2 days of contact with fresh tofu (“humid”) and after 7 days (“rancid”). The appearance of the “humid” defect is likely attributable to the migration of water and tofu odorants into the oil. The decline in the fruitiness intensity may result from the dissolution of volatile compounds associated with fruitiness in water or their interactions with tofu components such as proteins and carbohydrates. According to EU Regulation 2022/2104 [27], the median score for undesirable sensory properties for a high-quality category (extra VOO) must be zero. Consequently, all samples other than the control would fail to meet this criterion and would instead fall into a lower quality category (VOO).

## 3. Materials and Methods

### 3.1. Materials

A single production lot of prepacked tofu (Spar, Salzburg, Austria) was purchased from a local supermarket in Rijeka, Croatia. The average labeled nutritional composition of the tofu was as follows: 7.1 g fat (of which 1.2 g saturated fat), 0.5 g carbohydrates (of which 0.5 g were sugars), 3.2 g fibers, 12 g protein, and 0 g salt. The extra VOO (quality indices are presented in Table 3—control sample), produced during the 2023/2024 crop year, was purchased from Family Agricultural Holding Bellé Ervin (Buje, Croatia).

### 3.2. Sample Preparation

Fresh tofu was drained and cut into cubes with dimensions 1.5 cm × 1.2 cm × 1.2 cm. The fresh tofu cubes were weighed (60.3 ± 0.3 g) into sterilized glass jars (170 mL) and filled with VOO (100.2 ± 0.2 g). The jars were sealed with metal caps and stored in a refrigerator at 6 ± 2 °C in darkness for 1, 3, 5, and 7 days. Tofu/VOO samples were prepared in triplicates for each defined time point of the experiment. At the end of the contact time, tofu/VOO samples were allowed to reach room temperature and then gently shaken to homogenize the jar contents. VOO was separated from the tofu using a plastic sieve. The separated VOO was centrifuged at 6000 rpm for 5 min (centrifuge model EBA 200; Hettich, Tuttlingen, Germany) to remove residual tofu particles and water prior to performing chemical analyses.

For sensory analysis of VOO, an additional set of 5 jars of fresh tofu/VOO were prepared and stored under refrigeration at 6 ± 2 °C for 2 or 7 days. VOO separated from these jars was combined into a dark glass bottle and subjected to sensory analysis. Untreated VOO served as the control sample for both sensory and chemical analyses.

FD-T was prepared as follows: fresh tofu cubes (1.5 cm × 1.2 cm × 1.2 cm) were arranged in batches of 60 g on stainless steel shelves and frozen at −20 °C before freeze-drying for 48 h. Freeze-drying was performed using a freeze-dryer (model LIO-20 FP, Kambič, Semič, Slovenia) starting at −15 °C with a gradual temperature increase every 2 h until a final temperature of 40 °C was reached. FD-T obtained from 60 g of fresh tofu weighed, on average, 16.7 ± 0.5 g. These were transferred into sterilized glass jars (170 mL) and filled with VOO (100.2 ± 0.2 g). Samples were prepared in triplicates and stored under the identical conditions and time intervals as the fresh tofu/VOO samples. VOO was separated from FD-T and prepared as described for the fresh tofu/VOO samples.

### 3.3. Chemicals and Reagents

The following solvents were used in the analyses: ethanol 96% (UV-IR-HPLC grade), diethyl ether, n-hexane, and isooctane (purchased from Carlo Erba, Val de Reuil, France). Caffeic acid (purity 99%) and ethyl acetate were obtained from Panreac (Barcelona, Spain). Methanol and acetonitrile (both HPLC-grade), phosphoric acid, Fast Blue BB hemi (zinc chloride) salt with a dye content ≥ 80%, and phenolic standards (oleacein, oleocanthal, (+)-pinoresinol) were purchased from Sigma-Aldrich, Merck (Steinheim, Germany). Additional phenolic standards, including hydroxytyrosol, tyrosol, vanillic acid, vanillin, luteolin, and apigenin were purchased from Extrasynthese (Genay, France). All phenolic standards had a purity of 90% or higher.

### 3.4. Determination of Water Content in Tofu Samples

The water content of T and FD-T was analyzed in triplicate using moisture analyzer (HE 73, Mettler Toledo, Zurich, Switzerland) at 104 °C.

### 3.5. Determination of VOO Quality Indices

The free fatty acids (FFAs), peroxide value (PV), and spectrophotometric indices (K_232_ and K_268_) of VOO samples were determined according to the International Olive Council (IOC) analytical methods [29,30,31].

### 3.6. Extraction of Phenolic Compounds from VOO and HPLC-UV/VIS Analysis

Phenolic compounds were extracted from 2.5 g of VOO applying the IOC method [32] with SPE diol-bonded phase cartridges (6 mL/500 mg, Mecherey-Nagel, Düren, Germany). The extracts were evaporated using a rotary evaporator (RV 10 digital, Ika-Werke, Staufen, Germany) at room temperature. The dry residues were dissolved in 1 mL of methanol/water (1/1, *v*/*v*) and filtered through a cellulose acetate syringe filter (0.45 μm; Filtres Fioroni, Ingré, France).

Phenolic compounds in the extracts were analyzed using an HPLC Thermo Ultimate 3000 (Thermo-Fisher Scientific, Waltham, MA, USA) equipped with a UV/VIS detector capable of simultaneous measurement at 4 wavelengths. The method was used as described by Pasković et al. [33]. Separation of analytes was achieved using Lichrospher 100 RP-18 (250 × 4 mm, 5 μm) and a pre-column Lichrospher 100 (4 × 4 mm, 5 μm), both supplied by Agilent Technologies (Santa Clara, CA, USA). The mobile phase consisted of (A) 0.2% phosphoric acid and (B) methanol/acetonitrile (1/1, *v*/*v*). The flow rate was set at 0.8 mL/min at 25 °C. The solvent gradient was programmed as follows: 10% B 0–0.5 min; 10–16.5% B 0.5–25 min; 16.5–30% B 25–80 min; 30–100% B 80–95 min; 100% B 95–100 min; 100–10% B 100–102 min; and 10% B 102–105 min. This was followed by an additional 10 min equilibration period. The injection volume was 10 μL. UV/VIS detection was performed at 250 nm for vanillic acid; 280 nm for hydroxytyrosol, tyrosol, oleacein, oleocanthal, pinoresinol, and vanillin; 305 nm for apigenin; and 370 nm for luteolin. Stock solutions of the phenolic standards were prepared in methanol (80%, *v*/*v*), with the exception of oleacein and oleocanthal, which were prepared in acetonitrile. Phenolic compounds were identified by comparing their retention times with those of the standards and quantified by using the external standard method. Their concentrations were expressed as means of 3 sample preparations for each time point of the experiment in mg/kg of oil.

### 3.7. Determination of Total Phenols in VOO by Fast Blue BB Test

Total phenols were determined directly on VOO samples by applying a previously described method [7]. An aliquot of VOO (1.0–1.2 g) was weighed into a plastic test tube, and 2 mL of freshly prepared ethanol solution (70%, *v*/*v*) of Fast Blue BB reagent (0.1 *m*/*v*) and 2 mL of NaOH solution (5%, *m*/*v*) were added. The mixture was vigorously homogenized on a vortex apparatus with a test tube holder (Genius 3; Ika-Werke) for 20 min, followed by centrifugation at 6000 rpm for 5 min. The separated hydroalcoholic layer was transferred to another test tube and centrifuged again at 6000 rpm for 5 min. The absorbance of the resulting hydroalcoholic extract was measured at 420 nm using a UV/VIS spectrophotometer (DR/400, HACH, Loveland, CO, USA). Total phenols were expressed as mg of caffeic acid equivalents (CAE) per kg of oil, using 3 sample preparations for each time point of the experiment.

### 3.8. Determination of Total Phenols in Freeze-Dried Tofu by Fast Blue BB Test

FD-T cubes, separated from VOO, were defatted by successive extraction with 40 mL of n-hexane (4 times) and gentle shaking on a horizontal shaker (KS 130 Basic, Ika-Werke) for 10 min per extraction. The defatted FD-T cubes were air-dried and finely ground using a mortar and pestle. Phenols were extracted from 2.5 g of defatted FD-T using 20 mL of 70% ethanol (*v*/*v*) and mixing with a magnetic stirrer for 30 min. The extract was centrifuged twice at 6000 rpm for 5 min each time. The Fast Blue BB test was prepared with 2 mL of extract, 2 mL of Fast Blue BB reagent solution (0.1 *m*/*v*), and 2 mL NaOH solution (5%, *m*/*v*). The reaction mixture was vigorously shaken on a vortex apparatus for 20 min, and its absorbance was measured at 420 nm. Total phenols in defatted FD-T were expressed as mg of CAE per kg of dry mass.

### 3.9. Sensory Analysis of VOO

Sensory evaluation of the control extra VOO sample and VOO samples that were in contact with fresh tofu 2 and 7 days was conducted by the Sensory Panel of the Institute of Agriculture and Tourism (Poreč, Croatia). The panel consisted of eight assessors (three male, five female, average age 40 years) who were trained and experienced in VOO sensory evaluation according to the IOC method and guidelines [34,35]. The assessors underwent continuous training to enhance their qualitative and quantitative evaluation skills in accordance with IOC standards. Internal quality control procedures [36] included regular performance monitoring of all panel members. The sensory panel has been recognized by the IOC since 2014 and is accredited for the sensory analysis of VOO [37]. Informed consent was obtained from all assessors prior to participation in the study.

For oil sensory evaluation, a quantitative descriptive analysis was applied according to the IOC method [34] with a slightly modified profile sheet with a 10 cm unstructured intensity rating scale (0 cm for no intensity perception and 10 cm for the highest intensity) for odor and taste attributes. The evaluation sheet was expanded to include taste attributes “sweet” and “astringent”. Additionally, overall sensorial quality (total sensory score) was evaluated using a numerical scale ranging from 1 point (lowest quality) to 9 points (highest quality). The total sensory score was calculated as the mean of 8 values given by assessors.

### 3.10. Statistical Analysis

The results of single and total phenols in VOO, standard oil quality indices, and total phenols in defatted FD-T were subjected to one-way analysis of variance (ANOVA) to determine statistically significant differences (*p* < 0.05) among the days of tofu–VOO contact. Homogeneity of variance was tested using the Brown–Forsythe test, and mean values were compared using Tukey’s honestly significant difference test for equal or unequal sample sizes. Statistical analyses were performed using Statistica v. 14.0.0.15 software (Stat-Soft Inc., Tulsa, OK, USA).

## 4. Conclusions

This study provides new insights into the impact of high-moisture and protein-rich food on the phenolic compounds of VOO as well as the sensory properties of VOO during seven days of contact. A strong decrease in the total phenols and single phenolic compounds (specifically, hydroxytyrosol, tyrosol, oleacein, and oleocanthal) was revealed. The release of water from the fresh tofu significantly contributed to this decline, as evidenced by the substantially smaller decrease observed with the FD-T. The trend and dynamics of the phenolic compound decrease in VOO in contact with the FD-T was closely aligned with the increase in the FD-T. The contribution of water varied among the single phenolic compounds. Hydroxytyrosol conjugated compounds were more prone to interactions with tofu components other than water compared to tyrosol conjugated compounds. However, the data on the amount of released water and phenol content in water as well as in fresh tofu would provide a more complete insight into the role of water in these phenomena, probably confirming the conclusions drawn. The degradation of the VOO quality in contact with fresh tofu was observed only in its sensory properties, with a marked intensity reduction in the fruitiness, bitterness, and pungency, as well as the appearance of undesirable “humid” and “rancid” sensory defects. Future research should focus on the interaction mechanisms between phenolic compounds and various tofu components and on strategies to maintain VOO quality under such conditions, which is essential for its culinary applications.

## Figures and Tables

**Table 1:** Mass fraction (mg/kg) of single phenolic compounds determined by HPLC-UV/VIS in VOO used for immersion of fresh tofu (T) and freeze-dried tofu (FD-T) under cold storage (6 ± 2 °C).

Phenolic Compound	Tofu Variant	Duration of Tofu–VOO Contact				
		0 Day	1 Day	3 Days	5 Days	7 Days
Hydroxytyrosol	T	5.18 ± 0.31 ^a^	2.36 ± 0.13 ^bB^	1.10 ± 0.08 ^cB^	0.80 ± 0.19 ^cdB^	0.66 ± 0.10 ^dB^
	FD-T	5.18 ± 0.31 ^a^	3.99 ± 0.10 ^bA^	3.07 ± 0.16 ^cA^	2.50 ± 0.16 ^dA^	2.30 ± 0.15 ^dA^
Tyrosol	T	9.13 ± 0.12 ^a^	3.62 ± 0.14 ^bB^	1.86 ± 0.07 ^cB^	1.56 ± 0.08 ^dB^	1.61 ± 0.12 ^dB^
	FD-T	9.13 ± 0.12 ^a^	7.28 ± 0.29 ^bA^	6.26 ± 0.25 ^cA^	5.64 ± 0.07 ^dA^	5.58 ± 0.22 ^dA^
Vanillic acid	T	0.23 ± 0.04 ^ab^	0.25 ± 0.02 ^aA^	0.21 ± 0.03 ^abA^	0.22 ± 0.02 ^abA^	0.20 ± 0.03 ^bA^
	FD-T	0.23 ± 0.04 ^a^	0.19 ± 0.04 ^abB^	0.17 ± 0.04 ^abA^	0.18 ± 0.04 ^abB^	0.15 ± 0.04 ^bB^
Vanillin	T	0.12 ± 0.02 ^a^	0.12 ± 0.01 ^aA^	0.14 ± 0.01 ^aA^	0.15 ± 0.02 ^aA^	0.14 ± 0.02 ^aA^
	FD-T	0.12 ± 0.02 ^ab^	0.13 ± 0.02 ^abA^	0.14 ± 0.01 ^aA^	0.12 ± 0.02 ^bB^	0.13 ± 0.01 ^abA^
Oleacein	T	84.41 ± 2.72 ^a^	52.41 ± 4.15 ^bB^	34.69 ± 2.22 ^cB^	31.21 ± 5.48 ^cB^	15.31 ± 2.46 ^dB^
	FD-T	84.41 ± 2.72 ^a^	63.95 ± 7.73 ^bA^	52.23 ± 9.54 ^bcA^	46.73 ± 5.78 ^cA^	39.99 ± 6.25 ^cA^
Oleocanthal	T	148.06 ± 5.29 ^a^	92.19 ± 7.49 ^bB^	57.71 ± 4.76 ^cB^	50.67 ± 8.98 ^cB^	27.40 ± 4.35 ^dB^
	FD-T	148.06 ± 5.29 ^a^	121.14 ± 13.93 ^abA^	103.14 ± 17.89 ^bcA^	95.65 ± 12.45 ^cA^	87.99 ± 12.56 ^cA^
Luteolin	T	1.74 ± 0.03 ^a^	0.96 ± 0.04 ^bB^	0.65 ± 0.05 ^cB^	0.54 ± 0.06 ^dB^	0.42 ± 0.04 ^eB^
	FD-T	1.74 ± 0.03 ^a^	1.36 ± 0.03 ^bA^	1.23 ± 0.08 ^cA^	1.08 ± 0.07 ^dA^	1.00 ± 0.03 ^dA^
Apigenin	T	1.11 ± 0.04 ^a^	0.85 ± 0.01 ^bB^	0.70 ± 0.05 ^cB^	0.61 ± 0.04 ^dB^	0.52 ± 0.04 ^eB^
	FD-T	1.11 ± 0.04 ^a^	1.01 ± 0.02 ^aA^	0.91 ± 0.06 ^bA^	0.90 ± 0.06 ^bcA^	0.83 ± 0.04 ^cA^
Pinoresinol	T	8.61 ± 0.09 ^a^	6.18 ± 0.19 ^bB^	4.89 ± 0.25 ^cB^	4.56 ± 0.22 ^cB^	4.08 ± 0.17 ^dB^
	FD-T	8.61 ± 0.09 ^a^	7.80 ± 0.14 ^bA^	6.82 ± 0.36 ^cA^	6.88 ± 0.23 ^cA^	6.93 ± 0.25 c^A^
∑ phenolic compounds	T	258.59 ± 7.92 ^a^	158.94 ± 11.69 ^bB^	101.94 ± 7.15 ^cB^	90.34 ± 14.88 ^cB^	50.35 ± 6.81 ^dB^
	FD-T	258.59 ± 7.92 ^a^	206.86 ± 21.63 ^bA^	173.98 ± 27.92 ^bcA^	159.67 ± 18.33 ^cA^	144.91 ± 18.52 ^cA^
hs factor	T	0.58 ± 0.01 ^abc^	0.58 ± 0.01 ^bcA^	0.61 ± 0.02 ^abA^	0.62 ± 0.03 ^aA^	0.56 ± 0.02 ^cA^
	FD-T	0.58 ± 0.01 ^a^	0.54 ± 0.01 ^bB^	0.51 ± 0.01 ^cB^	0.49 ± 0.01 ^dB^	0.46 ± 0.00 ^eB^

The results are given as mean value ± standard deviation of 3 sample preparations. Mean values labeled with different lowercase letters in the same row are statistically different (one-way ANOVA, Tukey’s test for unequal N, *p* < 0.05). Mean values of single phenols labeled with different uppercase letters in the same column indicate statistically significant differences between T and FD-T (one-way ANOVA, Tukey’s test for equal N, *p* < 0.05). 0 day—control extra VOO that was not in contact with tofu; h factor—ratio between hydroxytyrosol (hydroxytyrosol + oleacein + luteolin) and tyrosol conjugated compounds (tyrosol + oleocanthal + apigenin).

**Table 2:** Literature data on water solubility of single phenolic compounds and percentage reduction in the concentration of single phenolic compounds in VOO after the first day of contact with fresh tofu (T) and freeze-dried tofu (FD-T) under cold storage (6 ± 2 °C).

Phenolic Compound	Water Solubility (g/L)	Percentage of Change After the First Contact Day		
		T (%)	FD-T (%)	T/FD-T
Tyrosol	25.3 [18]	−60	−20	3.0
Hydroxytyrosol	50.0 [19]	−55	−23	2.4
Luteolin	0.0000025 [20]	−45	−22	2.0
Oleocanthal	0.400 [21]	−38	−18	2.1
Oleacein	0.100 [21]	−38	−24	1.6
Pinoresinol	0.031 [22]	−28	−9	3.1
Apigenin	0.00083 [23]	−23	−9	2.6
Vanillic acid	1.5 [24]	0	−20	0.0
Vanillin	11.02 [25]	9	10	0.9

**Table 3:** Quality indices of control VOO sample and VOO samples after 7 days of immersion of fresh tofu (T) or freeze-dried tofu (FD-T) under cold storage (6 ± 2 °C).

VOO Sample	Storage Days	FFAs (% Oleic Acid)	K_232_	K_268_	PV (meq 0_2_/kg)
Control	0	0.33 ± 0.01 ^a^	2.24 ± 0.02 ^a^	0.15 ± 0.00 ^b^	14.73 ± 0.15 ^a^
In contact with T	7	0.31 ± 0.01 ^b^	1.96 ± 0.02 ^b^	0.11 ± 0.00 ^c^	14.67± 0.06 ^a^
In contact with FD-T	7	0.32 ± 0.01 ^ab^	2.24 ± 0.03 ^a^	0.19 ± 0.00 ^a^	nd ^1^
Limits for extra VOO category [27]		≤0.80	≤2.50	≤0.22	≤20

Abbreviations: FFAs—free fatty acids; K_232_ and K_268_—specific extinctions at 232 nm and 268 nm, respectively; PV—peroxide value. The results are presented as mean value ± standard deviation of 3 sample preparations. Mean values labeled with different letters in the same column are statistically different (one-way ANOVA, Tukey’s test for equal N, *p* < 0.05). ^1^ Not determined.

## Data Availability

The data are contained within the article.
